# Experimental SARS-CoV-2 Infection of Elk and Mule Deer 

**DOI:** 10.3201/eid3002.231093

**Published:** 2024-02

**Authors:** Stephanie M. Porter, Airn E. Hartwig, Helle Bielefeldt-Ohmann, Jeffrey M. Marano, J. Jeffrey Root, Angela M. Bosco-Lauth

**Affiliations:** US Department of Agriculture, Fort Collins, Colorado, USA (S.M. Porter, J.J. Root);; Colorado State University, Fort Collins (A.E. Hartwig, J.M. Marano, A.M. Bosco-Lauth);; University of Queensland, St Lucia, Queensland, Australia (H. Bielefeldt-Ohmann)

**Keywords:** SARS-CoV-2, severe acute respiratory syndrome coronavirus 2, COVID-19, 2019 novel coronavirus disease, coronavirus disease viruses, respiratory infections, zoonoses, cervid, *Cervus canadensis*, coronavirus, Delta variant, elk, mule deer, *Odocoileus hemionus*, wildlife, viruses

## Abstract

To assess the susceptibility of elk (*Cervus canadensis*) and mule deer (*Odocoileus hemionus*) to SARS-CoV-2, we performed experimental infections in both species. Elk did not shed infectious virus but mounted low-level serologic responses. Mule deer shed and transmitted virus and mounted pronounced serologic responses and thus could play a role in SARS-CoV-2 epidemiology.

Natural infections and experimental studies have indicated that diverse species of mammals can be infected with SARS-CoV-2 (*1*). The angiotensin-converting enzyme 2 receptor of white-tailed deer is closely homologous to that of humans. Angiotensin-converting enzyme 2 modeling studies predict that cervids, including sika deer (*Cervus nippon*), reindeer (*Rangifer tarandus*), and Père David’s deer (*Elaphurus davidianus*), are susceptible to SARS-CoV-2 (*2*–*4*). White-tailed deer are susceptible to experimental infection with SARS-CoV-2, subsequently shedding infectious virus and infecting naive conspecifics (*5*–*7*). Surveillance studies have demonstrated SARS-CoV-2 infection in free-ranging and captive white-tailed deer in the United States and Canada, determined by viral RNA detection, antibodies to SARS-CoV-2, or virus isolation (*8*–*11*). After their displacement in humans, the Alpha and Delta variants of concern persisted in white-tailed deer populations (*12*,*13*). Because SARS-CoV-2 is likely to have repeatedly spilled over from humans to white-tailed deer and circulated within North America deer populations, we assessed susceptibility of elk (*Cervus canadensis*) and mule deer (*Odocoileus hemionus*) to the Delta variant of SARS-CoV-2. All animal work was approved by the Colorado State University (Fort Collins, Colorado, USA) Institutional Animal Care and Use Committee.

## The Study

We studied 6 weanling elk (all female) and 6 yearling mule deer (5 female, 1 male). Animals were procured from private vendors and group housed (3 individuals of the same species/room) in an animal Biosafety Level 3 facility at Colorado State University. Before study commencement, all animals were seronegative for SARS-CoV-2.

We passaged the Delta variant of SARS-CoV-2 (BEI Resources, National Institute of Allergy and Infectious Diseases, National Institutes of Health: isolate hCoV-19/USA/MD-HP05647/2021 [lineage B.1.617.2]) 1 time in Vero cells. We then sequenced the isolate by using a next-generation pipeline and detected a single synonymous consensus change at amino acid 410 in nonstructural protein 14 (C to T transition) (GenBank accession no. OR758451) (*14*). We then intranasally inoculated 2 animals/room with 3.7–4.5 log_10_ PFU of virus diluted in phosphate-buffered saline (confirmed by back-titration of inoculum on Vero cells); the third animal in each room served as a contact.

We assessed the animals daily for signs of clinical disease (e.g., lethargy, anorexia, nasal discharge, sneezing, coughing, and dyspnea). One mule deer (no. 3) was tachypneic and coughing at arrival, and clinical signs continued throughout the week-long acclimation period until euthanasia at 3 days postinoculation (dpi) according to the study schedule. Because that respiratory pattern was present before study commencement, we did not attribute it to SARS-CoV-2 infection. No other clinical signs were observed in any animal.

**Figure 1:**
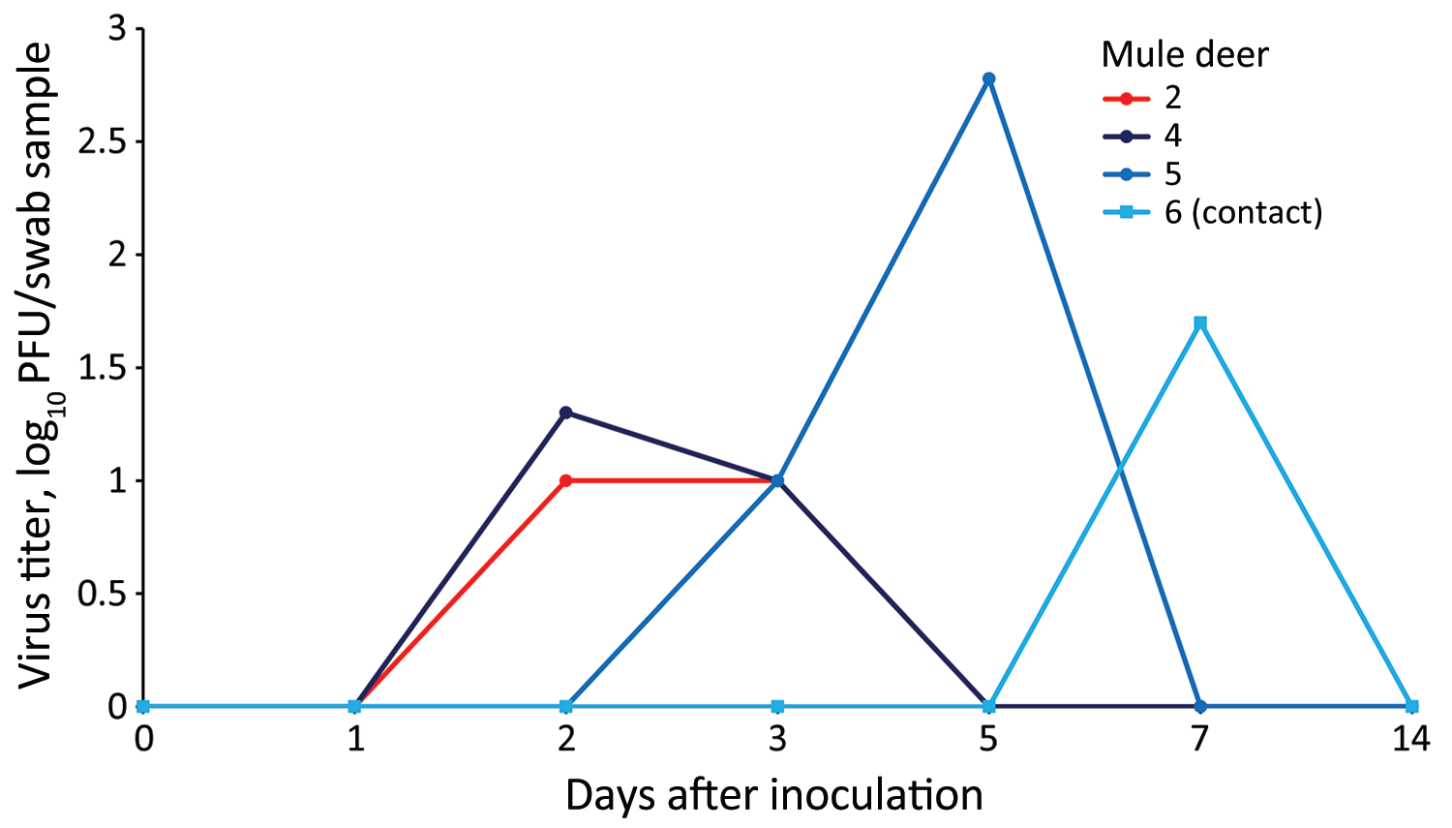
Oropharyngeal shedding of SARS-CoV-2 by experimentally infected mule deer as detected by plaque assay. Mule deer 2 was euthanized 3 days after infection. Mule deer 2, 4, and 5 were directly inoculated, and mule deer 6 was a contact animal.

**Figure 2:**
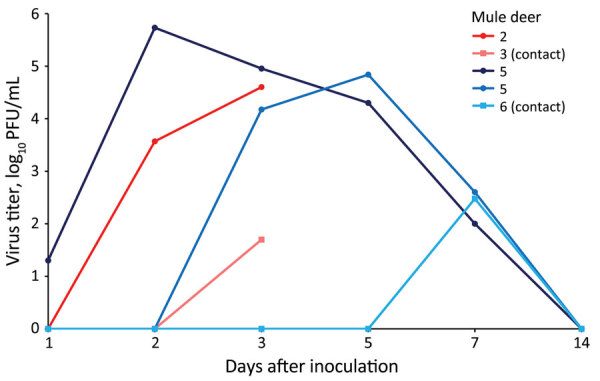
Nasal shedding of SARS-CoV-2 by experimentally infected mule deer as detected by plaque assay. Mule deer 2 and 3 were euthanized 3 days after infection. Mule deer 2, 4, and 5 were directly inoculated, and mule deer 3 and 6 were contact animals.

At 0, 1, 2, 3, 5, 7, and 14 dpi, we collected oral, nasal, and rectal swab samples. Because no elk shed infectious virus orally or nasally, we did not assess elk rectal swab samples. We performed reverse transcription PCR on elk oral and nasal swab samples collected through 7 dpi. We recovered SARS-CoV-2 RNA from samples from 3 directly inoculated elk collected at 1–5 dpi; cycle threshold values for all 3 were >28 (Table 1). Plaque assay showed that 3 of the 4 directly inoculated mule deer shed infectious virus orally and nasally (Figures 1, 2). Oral shedding of virus commenced at either 2 or 3 dpi and resolved by 7 dpi for directly inoculated mule deer. One contact mule deer shed virus orally at 7 dpi (Figure 1). Nasal shedding of virus was more staggered; inoculated animals initially shed virus at 1, 2, or 3 dpi, continuing through 7 dpi (in the 2 direct inoculants remaining at that time). For contact mule deer, only 1 nasal sample per animal was positive, collected at either 3 or 7 dpi (Figure 2). Infectious virus was not recovered from any mule deer rectal samples.

**Table 1:** PCR cycle threshold values for oral and nasal swab samples from elk experimentally infected with SARS-CoV-2*

Elk no. (infection route)	Oral swab sample, postinoculation day							Nasal swab sample, postinoculation day				
	0	1	2	3	5	7		1	2	3	5	7
1 (direct)	–	36.9	–	35.2	NA	NA		–	–	–	NA	NA
2 (direct)	–	–	–	–	NA	NA		35.6	35.0	–	NA	NA
3 (contact)	–	–	–	–	NA	NA		–	–	–	NA	NA
4 (direct)	–	–	–	–	–	–		33.6	29.9	34.4	26.5	–
5 (direct)	–	–	–	–	–	–		–	–	–	**–**	–
6 (contact)	–	–	–	–	–	–		–	–	–	–	–

*NA, not applicable because elk were euthanized on day 3; RT-PCR, reverse transcription PCR; –, no SARS-CoV-2 RNA was detected.

At 3 dpi, we euthanized and necropsied animals from 1 room of each species (n = 3; 2 inoculants and 1 contact) and collected tissues (nasal turbinates, trachea, heart, lung, liver, spleen, kidney, small intestine) for virus isolation and histopathologic examination. Although we did not detect infectious virus in any elk tissues, we recovered infectious virus from the nasal turbinates and trachea of 1 directly inoculated mule deer.

The remaining animals were maintained until 21 dpi, when they were euthanized and underwent necropsy with the same tissues collected into formalin. Blood from those animals was collected weekly and evaluated for a serologic response to SARS-CoV-2 by plaque reduction neutralization test (*15*). A low-level antibody response developed in both directly inoculated elk; peak neutralizing titers were 1:20 at 21 dpi. The contact elk did not seroconvert. Virus-neutralizing antibodies developed in all mule deer held until 21 dpi; peak titers reached >1:1,280 (Table 2).

**Table 2:** Plaque reduction neutralization test antibody titers from for elk and mule deer experimentally infected with SARS-CoV-2*

Animal	Preinfection	7 dpi	14 dpi	21 dpi
Elk 4	<10	<10	10	20
Elk 5	<10	<10	10	20
Elk 6	<10	<10	<10	<10
Mule deer 4	<20	40	5,120	1,280
Mule deer 6	<20	<20	1,280	1,280
Mule deer 5	<20	<20	1,280	640

*dpi, days postinoculation.

At necropsy, we observed no gross lesions in any animals. A veterinary pathologist evaluated all respiratory tissues from all mule deer. The tracheas of all mule deer were histologically unremarkable, but multifocal accumulations of mononuclear leukocytes in the absence of frank inflammation were noted in the nasal turbinates, lungs, or both from 5 mule deer. Elk tissues did not undergo histologic evaluation.

## Conclusions

If wildlife populations serve as maintenance hosts for SARS-CoV-2, the implications could be substantial. The persistence of virus variants already displaced in the human population, virus evolution, and spillback into a human have all been suggested to have occurred in white-tailed deer populations (*11*,*12*), although it is still unclear whether those deer will serve as maintenance hosts of the virus. Evaluating the susceptibility of other cervid species to SARS-CoV-2 will help direct surveillance efforts among free-ranging wildlife, which are key to understanding the epidemiology of SARS-CoV-2 and implementing control measures.

We used the Delta variant of SARS-CoV-2 to challenge animals in this experiment on the basis of evidence that this variant of concern was prevalent in white-tailed deer populations (*13*). Our results indicate that although elk seem to be minimally susceptible to infection with the Delta variant, mule deer are highly susceptible and capable of transmitting the virus. Inoculated elk showed no clinical signs, did not shed infectious virus, and mounted low-level humoral titers. The genomic RNA recovered from elk could represent residual inoculum. Infection in mule deer was subclinical, and although immune activation in the absence of frank inflammation was observed in respiratory tissues from 5 of the 6 animals, that finding may or may not be linked to SARS-CoV-2 infection. Of note, all mule deer used in this study were incidentally tested to assess their chronic wasting disease (CWD) status. Animals no. 1 and 3 were CWD positive, although it is unlikely that a concurrent infection with CWD greatly affected their susceptibility to infection with SARS-CoV-2 because only 1 of those animals became infected with SARS-CoV-2 while the other was the sole mule deer in our study that did not.

Experimental infection of elk and mule deer with SARS-CoV-2 revealed that although elk are minimally susceptible to infection, mule deer become infected, shed infectious virus, and can infect naive contacts. Mule deer are less widely distributed than white-tailed deer but still represent a population of cervids that is frequently in contact with humans and domestic animals. Therefore, susceptibility of mule deer provides yet another potential source for SARS-CoV-2 spillover or spillback. At this time, there is no evidence that wildlife are a significant source of SARS-CoV-2 exposure for humans, but the potential for this virus to become established in novel host species could lead to virus evolution in which novel variants may arise. Therefore, continued surveillance of species at risk, such as white-tailed and mule deer, is needed to detect any variants quickly and prevent transmission.
